# Discriminant Features and Temporal Structure of Nonmanuals in American Sign Language

**DOI:** 10.1371/journal.pone.0086268

**Published:** 2014-02-06

**Authors:** C. Fabian Benitez-Quiroz, Kadir Gökgöz, Ronnie B. Wilbur, Aleix M. Martinez

**Affiliations:** 1 The Ohio State University, Columbus, Ohio, United States of America; 2 Purdue University, West Lafayette, Indiana, United States of America; University Of Cambridge, United Kingdom

## Abstract

To fully define the grammar of American Sign Language (ASL), a linguistic model of its nonmanuals needs to be constructed. While significant progress has been made to understand the features defining ASL manuals, after years of research, much still needs to be done to uncover the discriminant nonmanual components. The major barrier to achieving this goal is the difficulty in correlating facial features and linguistic features, especially since these correlations may be temporally defined. For example, a facial feature (e.g., head moves down) occurring at the end of the movement of another facial feature (e.g., brows moves up), may specify a Hypothetical conditional, but only if this time relationship is maintained. In other instances, the single occurrence of a movement (e.g., brows move up) can be indicative of the same grammatical construction. In the present paper, we introduce a linguistic–computational approach to efficiently carry out this analysis. First, a linguistic model of the face is used to manually annotate a very large set of 2,347 videos of ASL nonmanuals (including tens of thousands of frames). Second, a computational approach is used to determine which features of the linguistic model are more informative of the grammatical rules under study. We used the proposed approach to study five types of sentences – Hypothetical conditionals, Yes/no questions, Wh-questions, Wh-questions postposed, and Assertions – plus their polarities – positive and negative. Our results verify several components of the standard model of ASL nonmanuals and, most importantly, identify several previously unreported features and their temporal relationship. Notably, our results uncovered a complex interaction between head position and mouth shape. These findings define some temporal structures of ASL nonmanuals not previously detected by other approaches.

## Introduction

Uncovering the grammar of sign languages is of fundamental importance in linguistics, cognitive science, education and engineering. Sign languages provide a window for the study of what formal, highly abstract and minimally required properties constitute human linguistic knowledge [1]–[3] *e.g.*, what is it about the human language system that makes it surface freely and in a full-fledged manner in the manual-visual modality when input from the oral-aural modality is not available [4]. Similarly, understanding how sign languages encode grammatical rules, which are thought to be rooted in the overall human cognitive capacity but which until recently were formally defined based mostly on spoken languages, allows researchers to generalize discoveries in the cognitive sciences [5]. Additionally, the teaching of sign languages will be much facilitated once we know more about how the grammar is encoded in its manual and nonmanual components in sign production at the clausal level. In sign language research, nonmanuals refer to linguistically-controlled uses of the face, head, and body other than the hands (see [6] for a recent review).

The sign language literature has made it clear that although affective and linguistic expressions may co-occur, they are nonetheless easily distinguished by their articulation onsets and offsets with respect to the signs made on the hands, with linguistic expressions tightly coordinated with the syntactic constituents that they relate to [6]–[18]. Similarly, there are clear distinctions between the nonmanual expressions and positions used by signers as compared to those employed by sign-naive hearing people in conjunction with speaking *e.g.*, [18]. It has been difficult to determine which facial expressions are associated with specific grammatical functions due to the fact that any given articulation could have meaning by itself or could enter into combination with other articulations to provide an unrelated meaning. The reason for this is related to the number of articulators (*e.g.*, head, brows, eye lids, eye gaze, nose, mouth, cheeks, chin, shoulders), the options available to each (for example, the head can turn left/right, nod up/down, or tilt left/right side), and the multiple combinations in which they interact.. Thus, sorting through all the possibilities and testing each for what may be subtle differences in meaning is a complex problem with many variables. While it is well known how the handshape, hand movement and palm orientation form the fundamental building blocks of the manual component of the sign [19]–[23], it is still unclear how head movements and facial configurations are structured and used in sign languages. Some progress has been made describing the nonmanual contribution based on, mostly, but not exclusively [6], [10], [13], [24], painstaking and slow annotation tools [4], [6], [9], [11], [12], [16], [25]–[27], but there is still much to be discovered about nonmanuals, especially with the help of more efficient research tools and procedures that are instrumentally-based and ideally automatic [28]–[32]. The development of computational approaches that can assist with this process will be of great benefit to linguistic analysis.

To better understand the need of computational tools for the linguistic analysis of nonmanuals, let us review their use in sign languages. The nonmanuals used in sign languages serve a variety of functions similar to those performed by intonation or word order changes in a spoken language like English. For example, to make a question from the English statement “Sarah is having a party this weekend,” the intonation pattern can be changed from falling at the end to rising at the end “Sarah is having a party this weekend?” (an echo question) or the word order can be changed to give “Is Sarah having a party this weekend?” (a yes/no question). To make similar questions, American Sign Language (ASL), like some spoken languages, does not use the option of changing the word order but instead adds nonmanual markers. In this example, the nonmanual marker is that of a “Yes/no question.” Such a marker is used to denote questions that can be readily answered with a simple “yes” or “no.” This is in contrast, for instance, to “Wh-questions” which start with a “wh”-word (or historical variant “h”) such as “which,” “when,” “how,” etc; in ASL Wh-questions are made with both the addition of nonmanual markers and optional word order changes. But each of these markers, for “Yes/no question” or “Wh-question,” may consist of multiple articulations, the most prominent being the position of the eyebrows, but with secondary articulations that may turn out to have their own meanings which combine with the primary meaning, or that may have emphasizer effects on the primary meaning, or that may be signer-specific, or even accidental and irrelevant [12]. When these functions are combined with the possible articulations and efforts to generalize to signer-independent patterns, the problem quickly becomes intractable.

**Figure 1:**
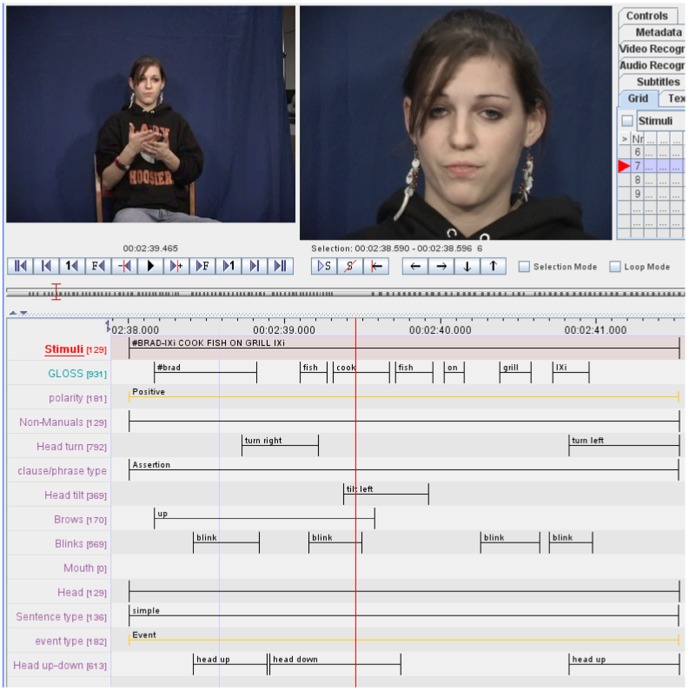
ELAN is a computer software that allows users to view synchronized videos simultaneously and frame by frame (top of figure), facilitating manual annotations (bottom half). Our manual annotations specify where the sentence starts and ends, where each word (or concept) starts and ends, plus the shape and configural features used to uncover the linguistic model (see text).

To identify nonmanual markers, sign language researchers will typically manually annotate head movements and facial expression changes observed in a large number of video sequences. Tools such as ELAN [33] have been specifically designed for this purpose, Fig. 1. ELAN allows visual observation of the starting and ending frame of the video sequence for each of these manual annotations. Furthermore, ELAN is a powerful tool that allows extracting data depending on the tiers, signed sentences, type of clauses or references over an interval of time among others. However, the aforementioned tool is not designed to perform statistical analysis and pattern recognition algorithms over the previously manually marked data. For this reason, analysis about the annotations is typically performed through a careful visual analysis to identify co-occurring nonmanuals and grammatical markers in large numbers of video sequences.

To date, research in ASL has identified that Hypothetical conditionals, Yes/no questions and Wh-questions are marked primarily by nonmanuals and secondarily by optional signs (*e.g.*, for conditionals, a sign with the meaning ‘if’ may be used but is not required) [34]. It has also been hypothesized that Wh-questions which have word order changed with the Wh-word moved to the end (“postposed”) could involve other distinct nonmanuals than those than those used in ordinary Wh-question [35]–[37]. Moreover, polarity (*i.e.*, positive versus negative) seems to be marked with nonmanuals; there is no regular sign for indicating positive polarity as this is the default interpretation in all languages, and negative signs for negative polarity are optional if the nonmanual for negation is present [10], [11], [38]. Due to the slowness of the standard approach used by linguists, it is difficult to verify to what extent these results hold over a larger number of video sequences or signers. Thus, it is unclear whether these are the only (required) nonmanuals used in these sentence types.

The present paper describes a linguistic-computational approach to *automatically* finding discriminant nonmanual features from a set of annotated videos. This approach involves two steps: first the procedure is validated by comparing the results with known discriminative features, that is, those already identified by sign language linguists, and then additional discriminative features and temporal structures are provided to linguists for further investigation and interpretation. This means that some features are known at the outset, but most are uncovered by the computational algorithms defined in the present paper. Taken together, these discriminant features and temporal structures comprise an expanded linguistic model of the nonmanuals under study. To achieve this goal, videos are annotated using a linguistic/articulated model of the face. Then, a computer algorithm automatically identifies facial articulations that correlate with a grammatical marker but do not co-occur elsewhere. The algorithm finds single nonmanual markers, such as a single facial component (*e.g.*, brows up), and first-order co-occurrences (i.e., temporal structure), as, for example, one facial or head articulation occurring before another (*e.g.*, head turns right *before* brows move up). Note that the term “discriminant” goes beyond a characterization of the nonmanual. While characterization defines the production of a nonmanual, discriminant features are those produced during one grammatical construction (e.g., wh-question) but absent elsewhere. This proposed approach will thus be used to test the hypothesis that nonmanual markers discriminate among the following nine classes of sentences: Hypothetical conditionals, Yes/no questions, Wh-questions, Wh-questions postposed, Assertions and their polarities (positive and negative).

This proposed approach not only validates some known nonmanuals but, most importantly, identifies a large variety of previously unsuspected nonmanual markers for each of the nine sentence types of ASL considered in the present paper. For example, as expected, our results show a systematic relationship between eyebrow position and grammatical constructions. As predicted by previous literature, ‘brows move up’ is prominent in Hypothetical conditionals (89.1%) and Yes/no questions (92.3%). Similarly, ‘brows move down’ occurs systematically in Wh-questions (89.5%) and Wh-questions with the Wh-sign postposed (99.2%). However, our results reveal a complex interaction between head position and mouth shape that has not been previously reported in the literature. This finding is extremely relevant because it shows how co-articulations of facial components are employed as grammatical constructions and hence emphasizes the importance of complex interaction of nonmanual markers in sign language.

The results summarized in the preceding paragraph would have been difficult to attain using a visual analysis of manual annotations. In contrast, the proposed computational approach can search for all possible first-order feature relationships and calculate which consistently co-occur in a given grammatical construct but rarely happen elsewhere. The approach and algorithms described in this paper have been incorporated into ELAN and can hence be readily used by other researchers to replicate and expand on the results reported herein.

## Methodology

We investigate the role of nonmanuals in five (5) types of sentences: Hypothetical conditionals, Yes/no questions, Wh-questions, Wh-questions postposed and Assertions; in addition we consider their polarities: positive and negative. This yields a total of 9 classes because Yes/no-questions are neutral, meaning they cannot be associated with a specific polarity (although this does occur in some other languages, *e.g.*, spoken English and Turkish Sign Language both allow negative Yes/no questions [39], [40]).

### Database

We recorded fifteen (15) Deaf native users of ASL signing more than 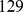 distinct sentences each [41]. Each of these sentences corresponds to the 9 classes (Appendix S1) defined above (*i.e.*, Hypothetical conditionals, Yes/no questions, Wh-questions, Wh-questions postposed, Assertions, and their polarities), for a total of 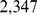 video sequences, although for variety of targets, not every signer produced exactly the same set of stimuli to incorporate variability in the data. This data variability is key to find generalizations of the model. For instance, we wish to see if the same discriminant temporal correlates are found in similar linguistic structures even when the productions differ; see Appendix S1 for lists of stimuli. It should be noted that signers were asked to replicate a series of sentences after watching video recordings of them. In this case, signers do not replicate the sentence (or group of sentences) exactly as in the video, but its meaning. Subject variability is expect and is indeed present in the collected dataset as was made clear after a careful analysis of each video sequence. Note that our goal is to use data with sufficient variability to allow us to recover the computational model of nonmanuals. This model can be put into test in subsequent field studies.

The signers were recorded using two high quality Sony DCR-VX2100 cameras. These cameras are equipped with 3 1/3″ CCDs for fast capture of color images in our studio conditions. All human subjects signed a consent form, granting permission for the use of their video sequences in research and the replication of these in scientific articles. The research and consent forms were approved by the IRB boards at The Ohio State University and Purdue University.

**Figure 2:**
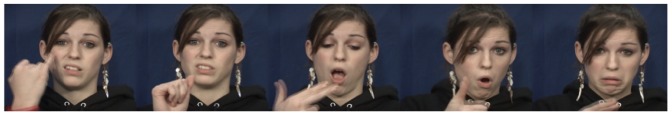
Samples of a video sequence of a native ASL signer signing “If #Sarah have a party tomorrow”.

The first camera recorded the upper-body (including the head) of the signer. The second camera captured a close-up of the face. This second camera provides high-quality video of the nonmanuals, Fig. 2. Watching both videos together, the sign language researchers manually labeled each video sequence as belonging to one of the five types of sentences listed in Appendix S1 and to one of their polarities. The sentences we consider are in Tables S1–S4 in Appendix S1 and the sentences signed by each one of the 15 participants in our database are in Table S5 in Appendix S1. These sentences correspond to 506 Hypothetical conditionals, 350 Wh-questions, 124 Wh-questions postposed, 313 Yes/no-questions, and 1,054 Assertions.

For consistency check, the annotations of each recorded sentence were visually validated by a native Deaf ASL signer and an experienced sign language researcher who were members of the American Sign Language Linguistics Laboratory at Purdue University. In particular, we made sure all video clips in the database correctly expressed its target sentence and that it was clearly visible and understood. Video clips not passing this test were eliminated from the database.

The video clips and manual annotations described in this section will be made publicly available to those wishing to extend on the results reported herein.

### Manual annotations

Research in face perception has demonstrated that facial expressions are coded and recognized by the cognitive system using configural [42] and shape [43] features. Configural refers to second-order changes. First-order changes code for the ordering of features (*e.g.*, nose on top of the mouth), while second-order specify between-feature distances. Shape features means that facial features are in a specified position (*e.g.*, the curvature of the mouth). These descriptions are correlated with facial movement that may also be defined using other coding systems [44].

**Figure 3:**
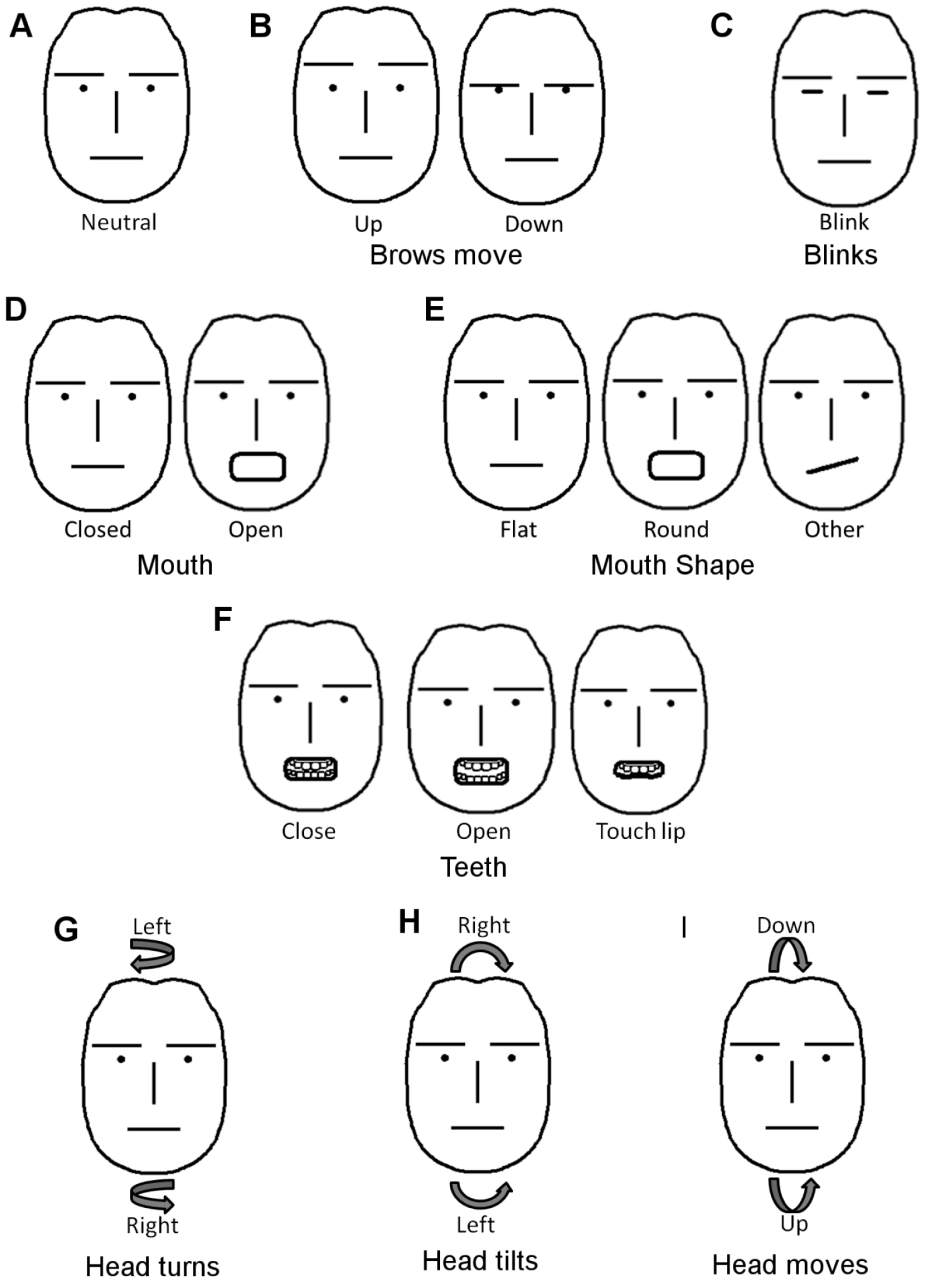
The configural and shape positions used to define each of the nonmanuals in sentences of ASL. In **A** we show a neutral face. A neutral face is defined as one without expression where all facial muscles are relaxed (except for the eyelids which are open). **B** We consider two configural positions for the eyebrows (up and down). **C** Blinks are marked by closing the eyelids. **D** The mouth can be open or closed. **E** We also annotate mouth shape where appropriate (flat, round and other). **F** When there is teeth showing, we consider three distinct positions – closed (top and bottom teeth touching), open (not touching), touching lips (where the top teeth are over the lower lip or the bottom teeth touch the upper lip). **G**–**I** We also consider the three possible rotations of the head – turns, tilts and forward/backward movements.

Similarly, sign language research has shown that such options as brow position, closed/open mouth and flat/round lips, teeth showing, and head turns are potential building blocks of nonmanual markers [18], [45]. We thus used fifteen (15) configural and shape feature positions corresponding to each of these nonmanual building blocks to annotate facial expressions in the video sequences of our database. These fifteen labels are summarized in Table 1 and Fig. 3.

**Table 1:** Features of the model and their entry sets.

Feature	Categories
Brows move	{Up, Down}
Blinks	{Blink}
Mouth	{Open, Closed}
Mouth shape	{Round, Flat, Other}
Teeth	{Closed, Open, Touch lip}
Head turns	{Left, Right}
Head tilts	{Left, Right}
Head moves	{Up, Down}

All video clips are displayed with the ELAN [33] software. A benefit of the ELAN software is that video sequences can be displayed frame by frame in synch with a time cursor so that the desired location for an event can be identified. A sign language expert can then manually annotate the configural and shape positions described above. This means that each annotation specifies where a configural or shape position starts and ends. An example of such a manual annotation is shown in Fig. 1. The manual annotations were reviewed by the two Purdue co-authors and, if necessary, changes were made until there was agreement in the coding.

The qualitative manual annotations described above must then be quantified in order to determine the most *discriminative* facial features. A possible solution is to treat a feature as a time varying function, where each category has some numerical value [28], [46]. The problem with this approach is that the sentences need to be aligned, that is, they must be shrunk or expanded to a canonical length. This would diminish or overemphasize some feature categories, especially those that expand a shorter time interval. Moreover, this approach would not model sequences of events, *e.g.*, headshakes, left to right turns, etc. We resolve these problems using Allen's Temporal Logic (ATL).

### Temporal logic description

**Figure 4:**
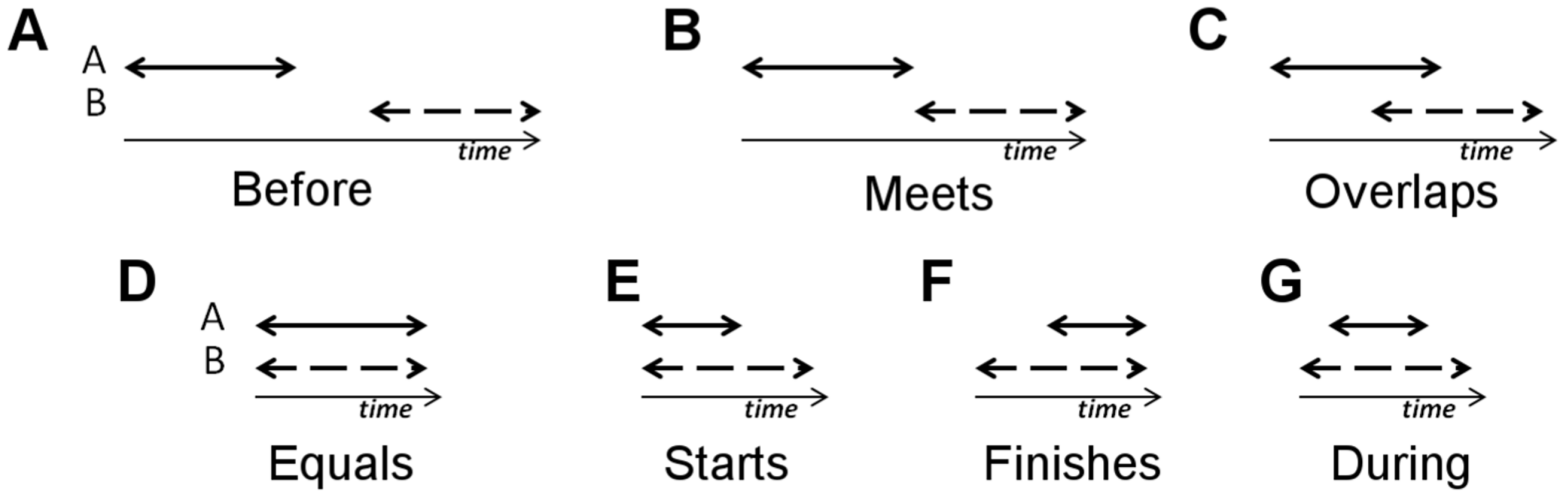
Visual representation of the propositions used in our coding.

ATL is a framework that allows us to analyze relative temporal information, such as *event A happens before event B* [47]. Here, any two time events are related by a set of symmetric, mutually exclusive binary relations, called propositions. In our modeling, we employ the following set of propositions: *before*, *meets*, *overlaps*, *equals*, *starts*, *during* and *finishes*. To show the use of the above defined propositions, consider the examples in Fig. 4. In this figure, we have two events, *A* and *B*. *A* is said to be *before B*, when *A* happens disjointly before *B*, Fig. 4.**A**. For example, *A* could be head turns right and *B* head turns left. Here, we would write *head turns right before head turns left*. This could be the case when a subject is signing a negative statement with negation marked with a headshake.

In the case that *A* happens immediately before *B*, then *A* is said to *meet B*, Fig. 4.**B**. Note that the difference between *before* and *meets* is that *before* requires a non-empty time interval between both events. For example, when nodding, the head moves up and down without a visual pause, which could be written as, *A meets B*. Obviously, in practice, two events involving different articulations would only perfectly follow one another by chance. To accommodate for small natural variabilities (*e.g.*, those due to data acquisition or small variations of the natural human movement between different subjects), we define *meets* as *B* occurring after a very brief interval 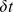 after *A*. The value of 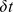 will be estimated using cross-validation in learning. In cross-validation, we divide the training data into two or more sets; use all but one of those sets for training while using the left out set for testing values of 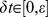, with 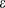 small. This is repeated multiple times to determine the value of the parameter yielding better generalizations. This is a common practice in pattern recognition where a learning algorithm uses a training set to come up with a representation that accurately represents some observations or discriminates between observations belonging to different categories (classes). A testing set is then used to determine whether the learned representation is capable of discriminating previously unseen examples into the correct class.

*A* is said to *overlap B* when *A* starts before *B* and *A* finishes during *B*, Fig. 4.**C**. In contrast, *equals* means that both events, *A* and *B*, share the same time interval, Fig. 4.**D**. This proposition is useful to denote single featural events, *e.g.*, to indicate that the brows move up once, as in Yes/no questions [34]. Although this may seem redundant at first, this notation allows us to consider single actions without changing notation or the algorithm.

**Figure 5:**
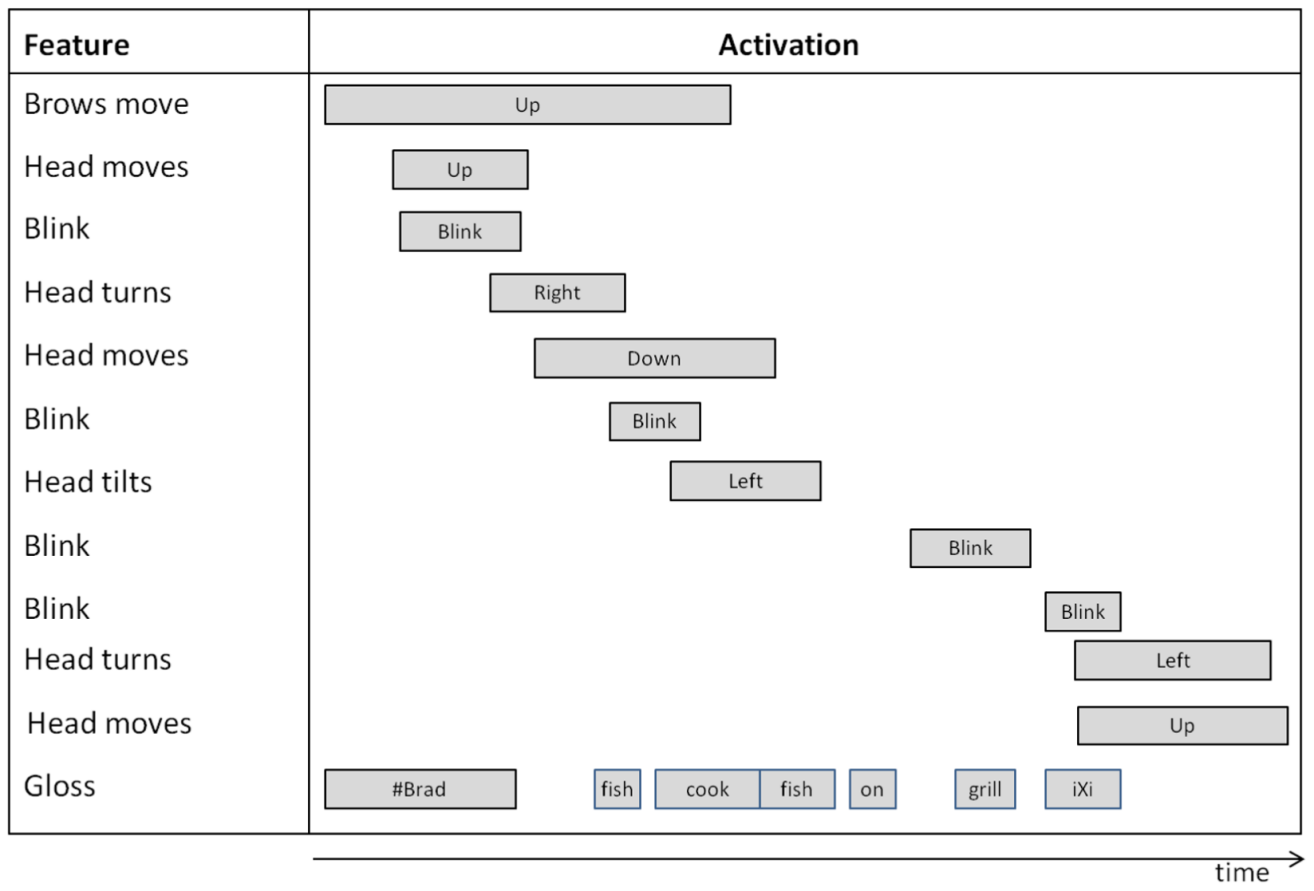
A diagram describing facial feature movements/positions and the gloss for the sentence “#BRAD-IXi COOK FISH ON GRILL IXi.” For clarity, here, we have listed the events and their time intervals in order of occurrence. The top row specifies the first event, with subsequent rows listing later occurring events. The bottom row summarizes the time interval of each signed concept. This visualization facilitates the coding of the events using the propositions in 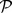. For example, for the figure above, it is easy to see that *head moves up* occurs *during* event *brows move up*, which can be compactly expressed as 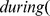
head moves up, brows move up).

When both events start at the same time but *A* finishes before *B*, then *A* is said to *start* with *B*, Fig. 4.**E**. Similarly, when events *A* and *B* finish at the same time but *A* starts after *B*, then *A* is said to *finish* at *B*, Fig. 4.**F**. Finally, *during* means that *A*'s time interval happens within *B*'s time interval, Fig. 5.**G**.

Fig. 5 shows an equivalent time diagram for the manual annotation previously illustrated in Fig. 1 for the sentence “#BRAD-IXi COOK FISH ON GRILL IXi,” (*i.e.*, “Brad is cooking/cooks fish on the grill”). The resulting coding using ATL relations is shown in Table 2.

**Table 2:** Temporal relations for the events in Fig. 5.

Event	Before	Meets	Overlaps	Equal	During	Starts	Finishes
Brows move up	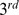 Blink, 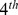 Blink, Head turns left, 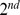 Head moves up	-	-	Head moves down, Head tilts left	Brows move up	-	-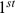 Head moves up	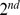 Blink, Head tilts left, 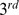 Blink, 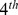 Blink, Head turns left, 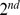 Head moves up	Head moves down	Head turns right	1st Head moves up	Brows move up	-	-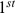 Blink	Head moves down, 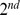 Blink, Head tilts left, 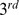 Blink, 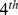 Blink, Head turns left, 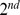 Head moves up	-	Head turns right	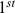 Blink	Brows move up, 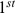 Head moves up	-	-
Head turns right	Head tilts left, 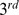 Blink, 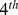 Blink, Head turns left, 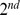 Head moves up	-	Head moves down, 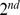 Blink	Head turns right	Brows move up	-	-
Head moves down	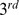 Blink, 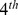 Blink, Head turns left, 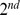 Head moves up	-	Head tilts left	Head moves down	-	-	-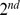 Blink	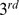 Blink, 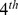 Blink, Head turns left, 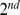 Head moves up	-	Head tilts left	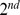 Blink	Brows move up, Head moves down,	-	-
Head tilts left	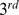 Blink, 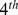 Blink, Head turns left, 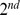 Head moves up	-	-	Head tilts left	-	-	-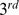 Blink	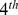 Blink, Head turns left, 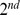 Head moves up	-	-	3rd Blink	-	-	-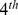 Blink	-	-	Head turns left, 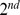 Head moves up	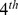 Blink	-	-	-
Head turns left	-	-	-	Head turns left	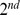 Head moves up	-	-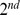 Head moves up	-	-	-	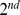 Head moves up	-	-	-

It is important to note in the table above that the temporal information is encoded in the description of the ATL using the propositions in 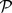. The temporal information is hence intrinsically coded in this table.

In summary, the Allen's Temporal Logic defined above is composed of a set of binary propositions. Formally, we denote this set as 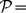
{before, meets, overlaps, starts, during, finishes}. The set 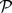 operates over the time interval defined by the set of events 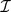. Therefore, an ATL can be formally denoted as ATL(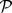, 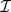). In this notation, any two events 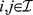 are related using one of the propositions in 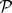, *e.g.*, 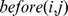 specifies that event 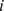 happened before event 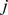.

The 17 feature categories (Table 1) form a set of 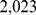 possible ATL first-order relations. We eliminated relations that cannot co-occur due to their mutually exclusive nature, (*e.g.*, brows move up *equals* brows move down) giving a total 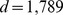 feasible relations.

It is also important to encode the number of consecutive occurrences for a given ATL relation. This might be important for some discriminant features, *e.g.*, while a single headshake may not carry any grammatical meaning, multiple headshakes can be a marker of negation or Wh-questions [12]. To correctly represent this information, we encode the relative frequency of each occurrence in a histogram, which displays the number of times that a given event happens.

**Figure 6:**
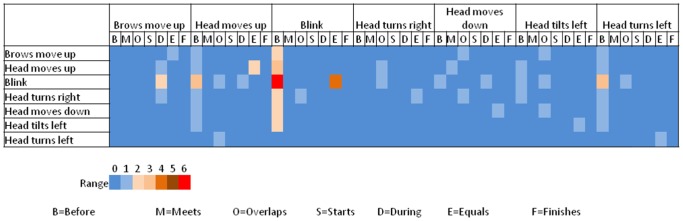
Visual representation of the ATL feature vector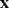. The dark blue color indicates a low number of occurrences for an event, while a dark orange color indicates a high number of repetitions. This figure is the histogram corresponding to the example in Fig. 5. The feature vector entries (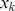) are read from left to right (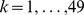).

Formally, we represent a sentence as 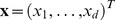, where 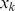 is the number of times that the first-order relation 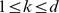 repeats in a sentence. For instance, if a sentence includes four eye blinks, the feature vector 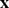 will have a value of 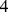 in the position 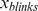; where we have used 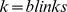 to indicate that this is the feature used to code for blinks. Fig. 6 shows the histogram for the example previously shown in Table 2 and Fig. 5.

### Discriminant analysis

The histogram representation of the ALT described thus far provides a convenient numerical representation of the nonmanual events we wish to study. To determine the time relations that best discriminate a grammatical structure from the rest (*e.g.*, Yes/no-questions versus the others), we need to use a feature extraction algorithm that uncovers the features or combinations of them that best *discriminate* between sentence types. In pattern recognition, such approaches are called discriminant analysis [48]. When the number of samples (relative to the number of features) is small, as is the case in the present study, Regularized Linear Discriminant Analysis (RLDA) is a possible algorithm to use [49]. RLDA adds a regularizing factor to the metrics being computed, preventing singularities even when the number of samples is small or when the underlying metric cannot be fully estimated [50]. Also, RLDA has a single parameter to estimate, making it very efficient and easy to work with [49].

Formally, RLDA finds the projection vector 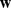 that best separates (in the least-square sense) two classes by maximizing the ratio between the class means to the average variance of these classes. Consider the case where 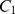 and 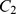 represent class 1 and 2, respectively. And, let the sample sets be 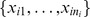, where 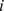 specifies the class and 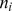 the number of samples belonging to it. The discriminant hyperplane separating the samples of these two classes is defined by its normal vector, 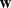. This vector is given by, 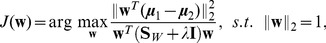
(1)where 
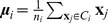
 are the sample class means, 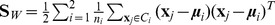 is the sample within-class scatter matrix, 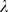 is the regularizing parameter that is found using cross-validation, 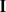 is the identity matrix and 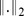 specifies the 2-norm (euclidean) measure. Recall that the regularizing parameter is used to ensure the above equation has a robust solution when the number of samples is small (*i.e.*, even if the within-class scatter matrix is singular).

Solving for (1) yields, 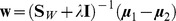.

An ATL relation is hence defined as *discriminative* if its corresponding absolute magnitude in 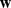 is larger than the others *i.e.*, 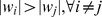. To rank their relative importance, each element of the vector 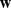 is normalized with respect to its largest attained value, *i.e.*, 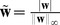 with elements 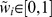, with 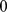 meaning the worst possible feature and 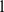 meaning the most important one, and where 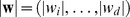, and 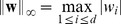.

Our *hypothesis* is that nonmanual markers can be used to discriminate among the nine classes of sentences described above. More specifically, we hypothesize that first-order temporal relations of facial movements are sufficient to code for such grammatical structure. To test this hypothesis, we use all the video sequences in our database except one to find the discriminant facial features (as described in the Methods section) and test whether the resulting model correctly classifies the left out sentence. This approached is known as Leave-One-Sentence-Out (LOSO) test.

Classification of the left out (test) sample 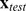 is done using the nearest-mean classifier. The nearest-mean classifier assigns to 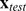 the class label 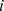 of the nearest class mean 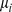, *i.e.*, 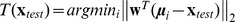. If we have 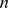 sample signed sentences, there are 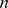 possible sentences we can leave out in the LOSO approach. In LOSO, we try all these 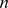 possibilities and then compute the mean classification accuracy. We also estimate the expected 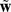 by averaging the 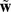 vectors generated from all LOSO iterations. Note that we only compute the classification accuracy for the features that provide the largest 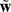, since this value is correlated with discriminability.

In addition to the above, we included the commonly used sensitivity index d′ to measure the distance between signal and noise for the most discriminative features. Here, d′ measures the performance of a single feature in isolation and, hence, does not provide information on co-occurring features or their temporal structures.

## Results

### Experiment 1: Constructions discriminant features

First, we wish to determine the nonmanuals that best discriminate each structure, *i.e.*, the discriminant features. To achieve this, we run a one-versus-all experiment. This means that, for each class (*e.g.*, Wh-questions), we use the linguistic-computational approach described in the Methods section to find the discriminant features that are common to that class but are not descriptive of the other classes.

The resulting discriminant features need to distinguish between the grammatical structures under study. These features are those providing the highest classification accuracies in the LOSO test described above. They are in Tables 3–7. The two columns in these tables labeled “% Activation” specify the characterization of the nonmanuals, i.e., the number of times the nonmanual is employed to marked a grammatical construction.

**Table 3:** Discriminant features for Hypothetical conditionals.

ATL relation	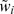	% Activation in Conditionals	% Activation in Others	d′	% Classification in Conditionals	% Classification in Others
Brows move up	1	89.1	54	1.13	89.1	46
Head moves down *finishes* brows move up	0.78	19	2.3	1.12	19	97.6
Head turns left *during* brows move up	0.67	50.4	13.6	1.11	50.4	85.2
Brows move up *equals* head moves down	0.65	18	2	1.14	18	97.9
Teeth touch lip *during* brows moves up	0.64	41.3	6.4	1.31	41.3	93.7
Mouth shape other *equals* mouth open	0.58	69	53	0.42	34.4	77.2
Teeth open	0.56	92.1	84.4	0.40	61.2	68
Mouth open *equals* brows move up	0.55	11.5	2.5	0.76	11.5	97.6
Teeth touch lip *during* mouth open	0.54	37.2	13.9	0.76	37.2	87.7
Head turns right	0.53	87	77.1	0.38	73.9	30.9

**Table 4 pone-0086268-t004:** Discriminant features for Wh-questions.

ATL relation	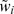	% Activation in Wh questions	% Activation in Others	d′	% Classification in Wh questions	% Classification in Others
Brows move up	1	10.6	70.5	1.79	89.4	73.6
Brows move down	0.99	89.4	23.2	1.98	89.4	62.4
Mouth shape round *starts* brows move down	0.7	43.1	0.5	2.44	43.1	99.3
Mouth shape flat	0.63	67.1	92.4	0.99	67.4	62.7
Mouth shape round	0.56	82.9	46.7	1.03	40.9	71.4
Head turns right *starts* brows move down	0.53	32	3.3	1.38	32	96.2

**Table 5 pone-0086268-t005:** Discriminant features for Wh-questions postposed.

ATL relation	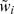	% Activation in Wh questions postposed	% Activation in Others	d′	% Classification in Wh questions postposed	% Classification in Others
Brows move down	1	99.2	29.4	2.95	99.2	64.7
Mouth shape other *overlaps* brows move down	0.68	43.5	4.9	1.50	43.5	94.6
Brows move down *finishes* mouth open	0.59	21.8	4	0.97	21.8	95.4
Blink *overlaps* brows move down	0.54	16.9	2.5	1.01	16.9	97.8
Mouth shape round *during* brows move down	0.5	61.3	9.5	1.6	61.3	87.2
Brows move up *meets* brows move down	0.49	37.1	5.1	1.30	37.1	94.8

**Table 6 pone-0086268-t006:** Discriminant features for Yes/no-questions.

ATL relation	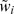	% Activation in Yes/no questions	% Activation in Others	d′	% Classification in Yes/no questions	% Classification in Others
Brows move down	1	6.1	37.3	1.22	93.9	58.6
Brows move up	0.81	92.3	56.8	1.26	92.3	46.8
Head moves down *starts* brows move up	0.71	35.5	7.9	1.04	35.4	93.3
Mouth shape flat *finishes* brows move up	0.55	32.9	3.4	1.38	32.9	97.2
Brows move up *before* brows move down	0.52	0	10.8	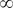	100	18.5
Brows move up *equals* mouth shape flat	0.51	30.7	3.9	1.26	30.6	96.5

**Table 7 pone-0086268-t007:** Discriminant features for Assertions.

ATL relation	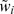	% Activation in Assertions	% Activation in Others	d′	% Classification in Assertions	% Classification in Others
Brows move down	1	17.1	46.2	0.85	82.9	55.9
Mouth shape round	0.61	39.9	61.9	0.56	62.6	63.3
Teeth close *during* brows move up	0.56	20.1	37	0.50	82.6	28.8
Brows move up	0.48	56.8	65.4	0.22	43.2	61.7
Mouth shape round *during* brows move down	0.46	3.2	19.6	0.99	96.8	27.6

In Tables 3–7 we also specify the classification accuracy of each of the discriminant features found with the proposed approach. To do this we use the following approach. Each discriminant feature 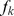 defines a one-dimensional feature space 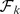 with its corresponding basis vector 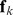. We project all vectors 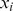 onto 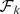, *i.e.*, 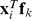. We then use RLDA to learn the hyperplane 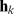 that best separates the samples of our two classes. Note that Linear discriminant analysis and RLDA provide the Bayes optimal solution when we have only two classes with equal variances [48]. Once this hyperplane has been determined, we compute the percentage of samples belonging to class 1 (*i.e.*, 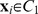) that are on one side of 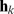 and the percentage of samples of class 2 (*i.e.*, 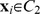) that are on the other side. These two numbers provide the percentage of classification accuracies listed in the last two columns in Tables 3–7.

The numbers in these last two columns (labeled “% Classification”) specify how many of our sentences can be correctly classified using each single feature 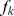. This refers to how *discriminant* the feature is. Some discriminant features will of course be more common and, hence, will successfully discriminate more samples of 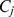 than others, with 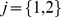. For example, “Head moves down *finishes* brows move up” in Table 3 is not a common nonmanual marker for Hypothetical conditionals (only used in 19% of Hypothetical conditionals), but it is almost never used elsewhere (2.3% of other sentences). This makes it a very efficient, robust stand-alone nonmanual to indicate a sentence is *not* a Hypothetical conditional (with classification accuracy at 97.6%). In comparison, “Brows move up” is a better nonmanual marker of conditionals (since 89.1% of our Hypothetical conditionals are successfully classified with it), but is also employed elsewhere (46.3% of other sentences are also classified as Hypothetical conditionals if one were to use only this feature). Thus, this second nonmanual is not as robust as the previous one. As expected, the result of averaging the last two columns in Tables 3–7 are highly correlated with d′. This is because both methods of analysis assume the data is Normally distributed. This correlation however is stronger for the single feature case, since d′ cannot account for temporal structure.

Additionally, we tested for the statistical significance of our results. This was done by comparing our results with those given by a randomization of the class labels. That is, we compare the results obtained with the proposed approach to the results one observes when the class labels for each of the samples 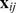 are assigned to a random class (rather than their true class label 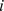). The randomization was repeated 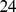 times, yielding a total of 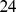 classification results. These results specify the probability of obtaining the classification accuracies by chance. A 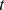-test of these revealed that our method performed significantly better than chance with the following 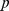 values: 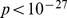 for Hypothetical conditionals, 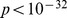 for Wh-question, 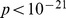 for Wh-questions postposed, 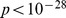 for Yes/no questions and 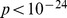 for Assertions.

Let us now describe the results of this study in detail for each of the 5 classes under consideration.

#### Hypothetical Conditionals

With respect to the Hypothetical conditionals (Table 3), the high percentage of “brows move up” is expected from the literature [16], [18], [34], [51], [52], as the conditional clause is routinely marked by raised brows. However, within the conditional clause, individual signs may require another facial posture that interferes with raised brows [13], and therefore not every sign in a Hypothetical conditional will have raised brows marked on it, thereby accounting for the less than 100% occurrence. For example, a facial expression that could interfere with the marking of conditional might be that of surprise, which involves brows up, head back, and eyes wide open. Furthermore, within the structures that are not Hypothetical conditionals (fourth column Table 3), there are Yes/no questions and topics in Assertions, which also are routinely marked by raised brows. Thus, 54% of the non-Hypothetical conditionals also show “brows move up.”

Most notably, Table 3 provides novel (and some unexpected) results concerning the behavior of the head, and the mouth and teeth. For instance “head moves down *finishes* brows move up” in 19% of the Hypothetical conditionals suggests a head thrust at the end of the conditional clause [53] and/or a prosodic reset [27], [35], [36] prior to the onset of the clause following the Hypothetical conditional clause.

Another frequent head behavior is “head turns left *during* brows move up,” which may reflect the establishment of a space to the left of the signer at head level to mark clauses containing content that is uncertain, hypothetical, or otherwise irrealis. The use of space for linguistic pragmatic functions has been recently reported for Catalan Sign Language (LSC) [54] and for Austrian Sign Language (OGS) [55]. Most relevant to the “head turns left *during* brows move up” in Hypothetical conditionals is Lackner's observation of the signers' reference to a “mental” space or “space of thoughts,” which may be coded by pointing, gazing up, or moving the chin up.

An additional head behavior, “head turns right,” raises another possible interpretation for “head turns left” in conditionals. As will be discussed in the Polarity section below, “head turns right *during* brows move up” occurs very frequently in clauses containing negation (negative polarity), as part of the negative headshake (right-left-right sequences [56]). Thus, the frequent occurrence (50.4%) of “head turns left *during* brows move up” in Hypothetical conditionals is highly associated with negation.

**Figure 7:**
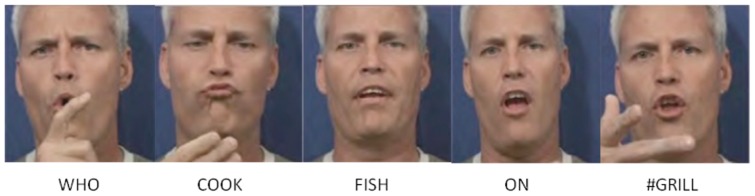
Mouth positions for the sentence “WHO COOK FISH ON #GRILL.”

Both Hypothetical conditionals and non-Hypotheticals have a high occurrence of “teeth open” in Table 3. For the Hypothetical conditionals, this is likely related to the frequent articulation of the word “if” when the sign IF is produced. This suggestion is strengthened by the more frequent occurrence of “teeth touch lip *during* brows move up” and “teeth touch lip *during* mouth open” in Hypothetical conditionals than in non-Hypotheticals – *i.e.*, the (upper) teeth touch the (bottom) lip at the end of the articulation of “if.” In contrast, the high frequency of “teeth open” in non-Hypotheticals is not accompanied by high occurrence of “teeth touch lip *during* brows move up” and “teeth touch lip *during* mouth open.” Instead, “teeth open” is the result of the inclusion of lexical items such as FISH in the stimuli. As reported in [57], nouns in ASL and other sign languages are much more likely to be accompanied by mouthing of the surrounding spoken language word than other word categories (*e.g.*, pronouns, verbs). Thus, it is not unusual that a noun sign like FISH would be accompanied by the articulation of “fish” or at least the first part of it that involves articulation of “f” or “fi.” Fig. 7 illustrates this in a sequence of mouth positions in one Wh-question produced by one of the ASL signers in our database.

#### Wh-Questions

From Table 4, we see that Wh-questions are separated from other constructions by both “brows move up” and “brows move down,” but in different ways. “Brows move down” is a well-known discriminant feature for Wh-questions in ASL [10], [51] and occurs in 89.4% of the Wh-questions in our sample. The occurrence of “brows move down” in 23.2% of the other constructions is likely related to the occurrence in those constructions of Wh-questions with the Wh-sign postposed (discussed separately in connection with Table 5). This is diminished when the downward movement of the brows is preceded by the head turning right

In contrast, “brows move up” occurs in few Wh-questions (10.6%) but is very frequent in other constructions (70.5%), which includes the Hypothetical conditionals discussed above and Yes/no questions (discussed below), both of which are associated with raised brows. “Brow move up” may also be associated with some occurrences of Wh-questions with Wh-sign postposed. This allows for very high classification rates of Wh-questions and other constructions even when they are using this single feature.

The remaining discriminative cue is “mouth shape round *starts* brows move down,” which occurs frequently in Wh-questions (43.1%) but not in other constructions (0.5%). This cue is likely associated with the presence of mouthing of “who” at the beginning of some Wh-questions. This is also the case for “mouth shape round.”

From the results in Table 4, we can thus identify a primary cue “brows move down” and a secondary cue “mouth shape round *starts* brows move down” for Wh-questions.

#### Wh-Questions postposed

A “Wh-question postposed” is one in which the Wh-word has been produced at the end of the question instead of at the beginning (described as “focus questions” in [27]). This placement of the Wh-word has the effect of allowing the main clause to be treated either as part of the question or as an Assertion followed by a question [58]. As a result, “brows move down” may cover the entire question or only the final Wh-word; either way, “brows move down” is a distinctive marker; Table 5. The occurrence of “brows move down” in other constructions is due to the inclusion of regular Wh-questions discussed above. When the signs preceding the postposed Wh-sign are treated as separate from the question at the end, we see very frequent (37.1%) “brows move up *meets* brows move down,” with the brows up on the non-question part and the brows down on the Wh-word. This “brows up *meets* brows down” pattern in ASL Wh-questions postposed is noted in [12] and discussed with respect to the presuppositional nature of the material preceding the postposed Wh-word in [37].

The mouth is also active in relation to “brows move down,” with “mouth shape round *during* brows move down” occurring in 61.3% of the Wh-questions postposed, as compared to only 9.5% in other constructions. Again, it is likely due to mouthing of “who,” which occurs frequently in Wh-questions postposed and also in regular Wh-questions which are included in the comparison constructions. “Mouth shape other *overlaps* with brows move down” frequently (43.5%) in Wh-questions postposed, and may be related to mouthing of other Wh-words, such as “which,” “why,” and “where.” Note the classification rate, for Wh-questions postposed and others is 94% when combining the features.

One articulation in Wh-questions postposed that did not show up in other constructions is the occurrence of blinks. “Blink *overlaps* brows move down” occurred in 16.9% of these as compared to only 2.5% in other constructions. Periodic blinks, the kind that are associated with eye-wetting, are well-known as a marker of the end of intonational phrases and syntactic constituents in ASL [14]. But if these blinks were just periodic blinks, they would occur after the brows move down ends. The fact that we see blinks overlapping with brows move down implies that they are deliberate blinks – slower and longer in duration. Deliberate blinks are associated with prominence on a sign [14]. If the blink ended at the same time as the brows move down, we would also know that the blink occurred on the last sign in the clause. The fact that blinks overlap with brows down means that the blink is located on a sign *inside* the clause. This supports the suggestion that they are deliberate blinks, which are used to emphasize a sign, because signs in final position in a clause are already emphasized/stressed [14] and therefore would not need a deliberate blink as a marker.

#### Yes/no Questions

Yes/no questions are distinguished primarily by “brows move up,” although this cue also occurs frequently in other constructions, which include Hypothetical conditionals (Table 6) and Assertions with marked topics. “Brows move up” and “brows move down” achieve very high classification accuracies for Yes/no questions – over 92%.

Note that, as expected, “brows move up *before* brows move down” does not occur in Yes/no questions, since the brow raise is expected to span the entire question [51]. In contrast, “brows move up *before* brows move down” does occur in other constructions, namely those in which a Topic or Hypothetical conditional clause (brows up) precedes a Wh-question.

“Head moves up *starts* brows move up” occurs in 33.5% of Yes/no questions but only 6.9% of other constructions. Half of the Yes/no questions are preceded by a topic; according to [59], two of the three possible topic markings involve head up. It is also claimed in [51] that head tilts forward with raised eyebrows in Yes/no questions. However, head behavior can also function parallel to body lean behavior, with tilt forward suggesting inclusion of the addressee and tilt back indicating exclusion of the addressee [18].

“Mouth shape flat *finishes* brows move up” occurs in 32.9% of the Yes/no questions as compared to only 3.4% of the other constructions, with a clear classification accuracy for the latter (97.2%). This is a truly surprising result which undoubtedly suggests further investigations in this direction as, to our knowledge, no function for flat mouth in ASL has been assigned in the existing literature. Since it spans the full duration of brows up (“brows move up *equals* mouth shape flat,” 30.7%) and ends when the brows up ends, these results suggest that this is a question mouth marker, although the issue is then raised as to why it is only not more frequent.

#### Assertions

Assertions have been traditionally viewed as not marked by specific nonmanuals, leaving the articulators free to reflect ones that accompany nonmanually marked lexical signs as well as to reflect the signer's emotional status. The cues identified as distinctive in Table 7 are notable for their relative absence in Assertions as compared to the other constructions. With respect to “brows move up,” the occurrence in Assertions is most likely due to the presence of topics with raised brows [59] prior to the Assertion itself.

### Experiment 2: Polarity discriminant features

The study of polarity follows the same procedure described above. The discriminant features selected by the LOSO approach are given in Tables 8–11. These are the results for each of the four classes with polarity, *i.e.*, Hypothetical conditionals, Wh-questions, Wh-questions postposed and Assertions.

**Table 8 pone-0086268-t008:** Discriminant features for polarity in Hypothetical conditionals.

ATL relation	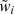	% Activation in Positives	% Activation in Negatives	d′	% Classification in Positives	% Classification in Negatives
Head turns left *before* head turns right	1	8.3	31.5	0.9	91.7	31.5
Head turns left *meets* head turns right	0.94	16.6	70.8	1.5	83.4	70.8
Head turns right *before* head turns left	0.59	7.9	27.8	0.8	92.1	27.8
Head turns right *during* brows move up	0.53	32.4	66.7	0.9	67.6	66.7
Head turns right *overlaps* mouth shape other	0.51	17.9	46.3	0.8	82.1	46.3

**Table 9 pone-0086268-t009:** Discriminant features for polarity in Wh-questions.

ATL relation	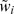	% Activation in Positives	% Activation in Negatives	d′	% Classification in Positives	% Classification in Negatives
Mouth closed *meets* teeth open	1.00	9.8	30.8	0.8	90.2	30.8
Teeth touch lip	0.89	25.2	6.7	0.8	25.2	93.3
Mouth shape other *before* head moves down	0.85	26.8	13.5	0.5	26.8	86.5
Mouth open *starts* brows move down	0.82	37.0	26.0	0.3	37.0	74.0
Teeth open *during* brows move down	0.82	39.8	63.5	0.6	60.2	63.5
Blink	0.76	69.5	76.9	0.2	76.8	46.2
Brows move down *starts* mouth open	0.74	8.1	15.4	0.4	91.9	15.4
Teeth open *overlaps* head turns right	0.74	11.0	30.8	0.7	89.0	30.8

**Table 10 pone-0086268-t010:** Discriminant features for polarity in Wh-questions postposed.

ATL relation	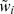	% Activation in Positives	% Activation in Negatives	d′	% Classification in Positives	% Classification in Negatives
Mouth shape other *before* mouth shape other	1	77.2	96.9	1.1	94.6	75
Mouth open	0.87	37	75	1.0	91.3	53.1
Head turns left *during* brows move down	0.87	46.7	96.9	1.9	94.6	65.6
Mouth open *before* mouth open	0.84	63	90.6	1.0	89.1	71.9
Head moves down *during* brows move down	0.84	45.7	75	0.8	54.3	75
Head turns left *before* mouth closed	0.83	28.3	71.9	1.2	90.2	43.8
Head turns left *during* mouth shape flat	0.78	3.3	37.5	1.5	96.7	37.5
Head tilts right *during* brows move down	0.77	13	59.4	1.4	87	59.4
Head turns left *overlaps* teeth open	0.75	15.2	53.1	1.1	84.8	53.1

**Table 11:** Discriminant features for polarity in Assertions.

ATL relation	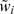	% Activation in Positives	% Activation in Negatives	d′	% Classification in Positives	% Classification in Negatives
Head turns left *before* head turns right	1	8.6	44.7	1.2	91.4	44.7
Head turns right *before* head turns left	0.97	10	43.6	1.1	90	43.6
Head turns right	0.97	62.4	97.2	1.6	96	68.9
Head tilts left *overlaps* head turns left	0.68	4.8	21.9	0.9	95.2	21.9
Head turns left	0.61	71.1	97.8	1.5	96	69.7
Brows move up *before* head turns right	0.52	1.6	20.8	1.3	98.4	20.8
Head turns right *meets* head turns left	0.51	13.9	69.7	1.6	98.4	48.6

Here, we also performed the statistical significant analysis described in Experiment 1 section. All our results were again statistically significant with: 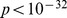 for Hypothetical conditionals, 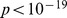 for Wh-questions, 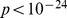 for Wh-questions postposed and 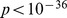 for Assertions.

Let us look at each of these results in more detail.

#### Hypothetical Conditionals

From Table 8, we see that all notable features for polarity in Hypothetical conditionals are associated with head turns and are more frequent in negatives than in positives. This is an expected finding as negatives are generally marked by headshakes in ASL [10], [11], [38] and many other sign languages [60]–[62]. As discussed earlier, “brows move up” is associated with Hypothetical conditionals, and the occurrence of “brows move up” with negative Hypothetical conditional head turns suggesting that both conditionality and negation can be distinctly shown simultaneously without interfering with each other [15].

When we dig into the details of the temporal behavior of head turns, we identify linguistic interactions that have not been available to impressionistic analysis so far. We believe this is an improvement our algorithm has made possible for sign language research. In this sense, the findings with the ordering and the relation of head turns alert us to two previously unrealized findings about negative polarity in ASL.

The first finding is that the defining relation for negative polarity is “a head turn *meets* the opposite head turn” which kinematically correlates to a fast paced headshake. That the defining relation is “meets” rather than a head turn preference on either side of the relation is proved when we compare Table 8 with Table 11. In Table 8, what gives us the fast paced headshake is the “head turns left *meets* head turns right” discriminant feature. On the other hand, what gives us the fast paced headshake in Table 11 is the “head turns right *meets* head turns left” discriminant feature. The commonality turns out to be the abstract linguistic relation “a head turn *meets* the opposite head turn.” The kinematic realization of this abstract linguistic property is a fast paced headshake.

The second finding is that we can generalize that negation normally begins with “head turns right.” Because this does not always occur, we state the general nonmanual marking as “a head turn *meets* the opposite head turn.” There is a widespread linguistic assumption that Assertions are the most basic, simplest clause type, and this is where we see the negative headshake start with “head turns right.”

When we look at the combination of Hypothetical conditional and negation, we are no longer looking at the simplest situation. Instead, the conditional contains the negation as part of its clause, and we expect the conditional marking to begin before the negation marking. In the case of constructions discriminant features for Hypothetical conditional, we determined that “head turns left *during* brows move up” is the discriminant feature for conditionals. As we will see in discriminant features for polarity in assertions, the primary indicator of fast paced negative headshake “head turns right *meets* head turns left” in Assertions, the most basic clause, starts with head turn to the right. In Hypothetical conditionals, the “head turns left” dominates the negative, and the fast paced negative headshake is modified to start on the left, yielding “head turns left *meets* head turns right”, the most active nonmanual marker in negative Hypothetical conditions (Table 8).

In addition to these two findings we also need to note that the headshakes reflected by “head turns right/left *before* head turns left/right,” with a short pause between the two, rarely occur in positive Hypothetical conditionals (7.9% and 8.3%), leading to 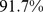 and 92.1% classification accuracy from the single feature of pausing alone. This observation supports our contention that assimilated “head turns left” starts the marking of negation and fast paced meeting of “head turns right” continues the marking. Without the “head turns right” as the second half of the fast paced negative headshake in positive conditionals, there is no purpose to the brief pause that separates the fast paced headshake from the rest of the head turns. Therefore, brief pauses between head turns highlights the separation of the fast paced negative headshake from the rest of the headshakes in negative conditionals. There is no need for these pauses in positive conditionals as the only head turns present are related to conditionality.

Beyond the results above, our results further highlight the role of the mouth in nonmanuals. Note the frequency of “mouth shape other” (meaning, not round or flat) during (overlapping with) “head turns right” in a large number of negative Hypothetical conditionals.

**Figure 8:**
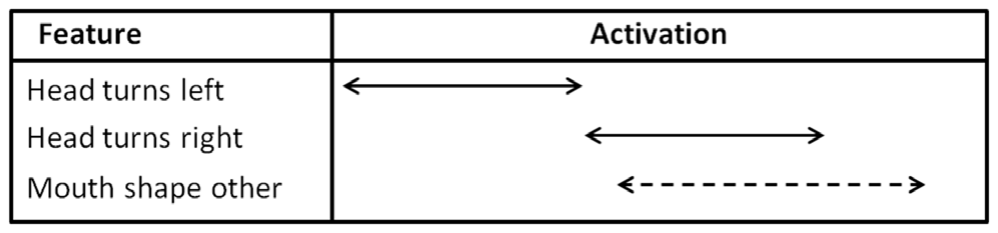
Computational model of polarity in Hypothetical conditionals.

As we noted above, the fast paced “head turns left *meets* head turns right” gives us a strong cue for differentiating negative polarity from positive polarity in the conditional sentences. When this result is evaluated with “head turns right *overlaps* mouth shape other,” we come up with the pattern in Figure 8 where “mouth shape other” overlaps with the second half of the headshake. This temporal relation gives us another interesting and novel finding in that “mouth shape other” temporally occurs after the onset of negation as marked by the first head turn to left. Although the involvement of the mouth for negation in ASL had been detected in previous research [11], given the technology of the time, back then it was only possible to report the timing relation between the headshake and the hand movement, but not the exact temporal relation between the two nonmanual markers headshake and mouth position.

Next, note that the percentage of “mouth shape other” (46.3% vs. 17.9%) is strong enough not to be associated with a combined effect of lexical mouth-shapes of random signs in negative sentences. The contrast in discriminant percentages indicates that the mouth is actively involved in the expression of negation in ASL. This finding (82.1% and 46.3%, respectively) is consistent with results reported in [11]. In their study, negative sentences were compared to positive controls; headshakes were not always present in negatives, and whether or not headshakes were present, there was involvement of the mouth and/or chin in 96.5% of the negative productions. Furthermore, they noted that the most frequent combinations of nonmanual markings for negatives involved eyes (squished or closed) and a mouth position (corners of mouth down, mouth stretched, mouth tightly closed, chin contracted). These mouth positions are included in our coding of “mouth shape other.”

The fact that “mouth shape other” is not as frequent as headshake is another interesting finding. There are two ways to interpret this finding. First, although “mouth shape other” is present for almost half of the negative sentences, it could be a redundant or secondary prosodic cue, similar to the findings in [63] where the non-dominant hand is considered a secondary cue with respect to the primary cue of change in the mouth area tension. Therefore, “mouth shape other” would not need to occur as frequently as headshake. In this sense, headshake alone would be a sufficient prosodic cue for introducing negative polarity in conditionals. Second, the presence of “mouth shape other” could be a primary cue parallel to headshake. However, the combined semantic effect of headshake and “mouth shape other” may be more emphatic than the headshake alone. Therefore, the combination would only occur in situations where emphasis needs to be cued while headshake is more persistently present as the primary negative cue. Both of these possibilities need to be tested. The first one may be tested through prosodic perception studies while the second possibility may be tested with a semantic interpretation study. The upshot of the contribution of the current study is that the algorithm used in this study makes it possible for us to voice these two possibilities due to the temporal and distributional accuracy that we attain.

#### Wh-questions

The discriminant features that distinguish negative and positive polarity in Wh-questions are more varied than those of Hypothetical conditionals and seem to be less clearly reflective of general negative marking. That is, they generally do not indicate head turns. Instead, a number of the features relate to mouth and teeth positions. In addition, there is no clear pattern of occurrence such as that seen with Hypothetical conditionals, where strong markings were seen for negatives as compared to positives. Here, sometimes a mouth or teeth feature is more prevalent in negatives and sometimes the reverse is true. This suggests that while Wh-questions can be clearly marked by brows down, when Wh-questions are negative, nonmanuals alone may not be able to carry both semantic functions. Such a conclusion is in keeping with two other observations in the literature. One is that both Wh-marking and negation use headshakes; the negative headshake is somewhat larger and slower [12]. The other is that whereas Yes/no questions rarely are marked by a manual sign and rely primarily on the brows up nonmanual marking, Wh-questions are most frequently accompanied by a manual Wh-sign. There are some notable examples where a Wh-question can occur without a Wh-sign, for example MANY “how many,” COLOR “what color” [64]. But reliance on Wh-signs means that nonmanuals may not be systematically recruited to carry the full load of semantic marking by themselves. These results suggest that when negative and Wh-questions interact, nonmanuals like the mouth become more important.

**Figure 9:**
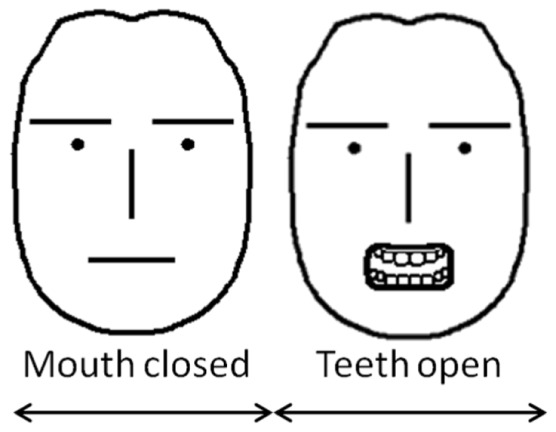
Computational model of positive versus negative polarity in Wh-questions.

Moreover, the current results define several interesting interactions in Wh-questions and polarity. “Brows move down *starts* mouth open” is highly classificatory (91.9%) for positive Wh-questions by its absence. While “brows move down” is clearly related to Wh-questions, mouth open could be related to some of the Wh-words being mouthed (*e.g.*, who, what, which, when, why, etc.) and given the higher occurrence in negatives, possibly also ‘not.’ Another mouth cue with a high classification value (89%) for positives by its absence, “teeth open overlaps head turns right,” is almost three times more prevalent in negatives than in positives. Similarly, “mouth closed *meets* teeth open” is twice as prevalent in negatives as in positives and has a high classification value – its absence from positives yields correct classification 90.2% of the time despite its rare occurrence in Wh-questions in general. When such negative evidence (9.8%) is combined with positive evidence (30.8%), we may thus suggest that “mouth closed *meets* teeth open” is a candidate to discriminate between negative and positive polarity in Wh-questions. The computational model of this interaction is given in Figure 9.

As we have discussed in the section above regarding negative conditionals, there is evidence of “mouth closed” as a marker of negation. The fact that it meets “teeth open” 30.8% of the time suggests that this cue may be interrupted by some lexical interference (mouthing of English words) tucked into the flow of prosody due to certain lexical items.

Another mouth feature that has a high classification value for negative Wh-questions is “teeth touch lip,” which occurs in 25.2% of positives versus only 6.7% of negatives. This is likely the result of three of the positive Wh-questions containing signs that can be accompanied by mouthing of English words beginning with ‘f’ (fish, forks, finish).

#### Wh-questions postposed

In contrast to regular Wh-questions, there is a clearer pattern to negative marking for Wh-questions postposed, with discriminant features all occurring more frequently in the negatives than in the positives. This pattern seems to support the argument above concerning regular Wh-questions. The basic difference between Wh-questions with and without Wh-sign postposing is that when the Wh-sign occurs at the end of the question, the material that occurs before the Wh-sign does not have to be covered by Wh-marking. As discussed in discriminant features for polarity in Wh-questions, the material prior to the Wh-sign can sometimes be considered an Assertion, meaning that Wh-marking and negation marking would not come into conflict. Hence, Table 10 reflects features of negation on non-Wh-marked signs. This means that the nonmanuals can carry negation clearly, as seen by the prevalence of head turns among the discriminant features. This suggests a fundamental linguistic difference between Wh-questions and Wh-questions postposed which confirms previous research [37].

Like regular Wh-questions, we see increased prominence of mouth and teeth positions which will require further research to explain, such as the interaction of mouth gestures with mouthing the English words when certain signs are produced [57]. Once again, this is an important, novel finding, reinforcing the previously overlooked suggestion of a more relevant mouth role in polarity [11].

#### Assertions

The results for polarity marking of Assertions also show clear nonmanual marking of negation, as all discriminant features occur more often in negatives than in positives. The primary cue in all discriminant features is head turn, reflecting negative headshakes. The only other discriminant cue is “brows move up,” which occurs before “head turns right;” this is the result of those Assertions that begin with a topic or are preceded by a conditional clause, both of which are marked with brows up, followed by a negative Assertion marked with headshake.

In sum, with the exception of Wh-questions, the marking of negative polarity is clear on the constructions included in this study, and Wh-questions themselves are known to differ from the other constructions in needing a manual Wh-sign most of the time. The surprises in the data are related to mouth and teeth positions, which seem to gain prominence as nonmanual marking becomes more complex when multiple semantic functions are expressed simultaneously.

## Discussion

Uncovering the discriminant features of the linguistic model governing nonmanuals in sign languages has proven to be an extremely hard problem. The present paper shows how this can be resolved using a linguistic-computational approach. In this approach a linguistic representation of the face is first obtained. A computational approach is then employed to determine the combination of these features consistently observed in each class but not with others. The resulting linguistic model proves to be able to discriminate between nine different classes of sentences – Hypothetical conditionals, Wh-questions, Wh-questions postposed and Assertions in their two polarities and Yes/no questions in positive polarity.

The analyses described above strongly suggest that there are discriminant features that can be used to separate conditionals from non-conditionals, Yes/no questions from non-Yes/no-questions, Wh-questions and Wh-questions with postposed Wh-signs from non-Wh-questions, and Assertions from non-Assertions. In addition, for each of these except Yes/no questions which do not form negative in ASL, the discriminant features separate the negative structures from their positive counterparts. From the model (Tables 3–11), the results indicate that some features are more relevant to accomplishing these distinctions than others. For example, blinks do not play a role in making these structural distinctions, nor was it expected that they would, as their function is more closely related to the marking of constituent structure (syntactic phrases) and the intonational phrasing that surrounds them [14]. Similarly, head tilts and head movements up and down appear to play no major role, leaving open the question of what their functions might be. Clearly the relevant features identified by these analyses are the head turns, brow positions, and mouth and teeth features. The results for brow position confirm our expectations, both for “brows move up” and “brows move down.”

In addition, the algorithm gives temporal relations that are striking with respect to head turns, where there are both expected and important novel results. The use of multiple head turns as headshakes has been well-documented for ASL and other sign languages as a major nonmanual marker of negation [10], [11], [38], [60], [62]. However, our findings with respect to temporal relations need to be emphasized because, as mentioned, although we know what makes a headshake, until now we did not have the means to quantitatively measure the temporal make-up of the interaction of the components of a headshake. In other words, the results of the present study suggest that not all temporal sequences of head turns left/right plus head turns right/left are the same. In negative conditionals and Assertions, negative polarity is most strongly cued when these meet one another, *i.e.*, a faster paced headshake.

This opens a new venue for the study of headshakes. For instance, with regard to negative head turns it will be important to determine whether all negative headshakes are faster under all conditions across multiple sign languages. This possibility is raised in observations on Austrian Sign Language [55] concerning faster headshakes on negatives that follow regular speed headshakes on conditionals. The analysis can also be expanded to investigate if there are quantitative differences between languages that use headshake as a primary nonmanual cue such as ASL as compared to those that use negation as a secondary cue in addition to a different major nonmanual marker, such as Turkish Sign Language [65]. We also expect that these two novel findings for ASL will urge researchers of other sign languages to quantitatively investigate the nature of headshake since the surface cue, *i.e.*, headshake, may very well be instantiated in more than one articulatory combination given the left and right directions of articulation, as well as temporal possibilities; a priori there is no reason to expect other sign languages which employ “headshake” as the major nonmanual cue to behave the same way as ASL does. On the big picture, this path also opens up an exciting agenda, both for ASL and cross-linguistic research, for quantitatively detecting nuances in the behavior of certain nonmanual markers which look the same on the surface even to the eye of an experienced sign language annotator.

In addition to the insights about negation reported here, the approach presented in the present work also revealed that head turn left is a discriminant feature in conditionals. Again, work on Austrian Sign Language [55] is relevant for furthering research on this finding, noting that signers who are talking about things they think or wonder about use a higher, right side space. Conditionals are just such a possibility, as they indicate not fact but possibility, a hypothetical thought, possibly placed on the right for Austrian Sign Language. Comparing our findings with these in [55] opens up a research domain for further investigating crosslinguistic similarities and differences with the use space for conditionals.

Lastly, the results also highlight the important role that the mouth and teeth play in negation. It is noted in [11] that the most frequent combinations of nonmanual markings for negatives involved eyes (squished or closed) and a mouth position (corners of mouth down, mouth stretched, mouth tightly closed, chin contracted). These mouth positions are included in our coding of “mouth shape other,” which shows up as a discriminant feature overlapping with head turns in negatives. As we discuss above, the involvement of the mouth and teeth suggests importance of investigations in wider linguistic context to tease apart the possible secondary cue of “mouth shape other” from a possible interpretation of it as having a primary but emphatic function. Thus, these findings allow us to set up future studies by identifying the relevant variables that need to be controlled.

As a final note, it should be noted that the methodology described herein (and the implementation of the computational approach in Elan) will most probably find applications beyond the studies of sign language. Elan is a generic tool used in several disciplines and the statstistical analysis described in the present paper is equally valid in these studies.

## Supporting Information

Appendix S1 **Supporting Tables S1–S5.**
(TEX)
